# Supplementary material for: Recently Evolved, Stage‐Specific Genes Are Enriched at Life‐Stage Transitions in Flies
Source: J Exp Zool B Mol Dev Evol. 2025 Jul 15;344(7):428–41. doi: 10.1002/jez.b.23317 (PMC12576388; doi:10.1002/jez.b.23317)
Supplement: Supplementary file 1 — Figure 1 Supplement 1.pdf. [file JEZ-344-428-s013.pdf]

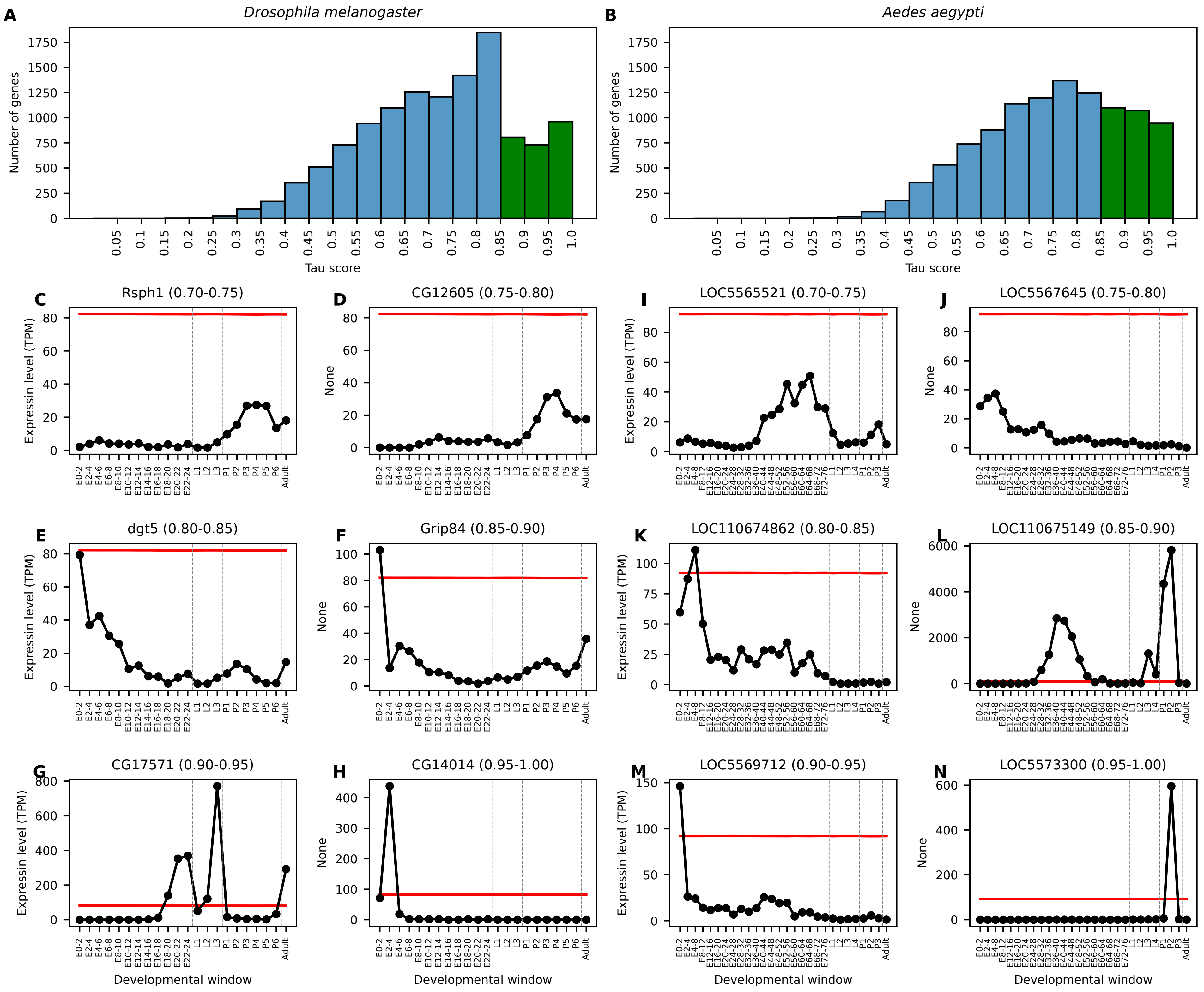
A
Drosophila melanogaster
B
Aedes aegypti
Number of genes
Tau score
C
Rsph1 (0.70-0.75)
D
CG12605 (0.75-0.80)
I
LOC5565521 (0.70-0.75)
J
LOC5567645 (0.75-0.80)
E
dgt5 (0.80-0.85)
F
Grip84 (0.85-0.90)
K
LOC110674862 (0.80-0.85)
L
LOC110675149 (0.85-0.90)
G
CG17571 (0.90-0.95)
H
CG14014 (0.95-1.00)
M
LOC5569712 (0.90-0.95)
N
LOC5573300 (0.95-1.00)
Expressin level (TPM)
None
Developmental window
