# Supplementary figures and images for: Recently Evolved, Stage‐Specific Genes Are Enriched at Life‐Stage Transitions in Flies
Source: J Exp Zool B Mol Dev Evol. 2025 Jul 15;344(7):428–41. doi: 10.1002/jez.b.23317 (PMC12576388; doi:10.1002/jez.b.23317)

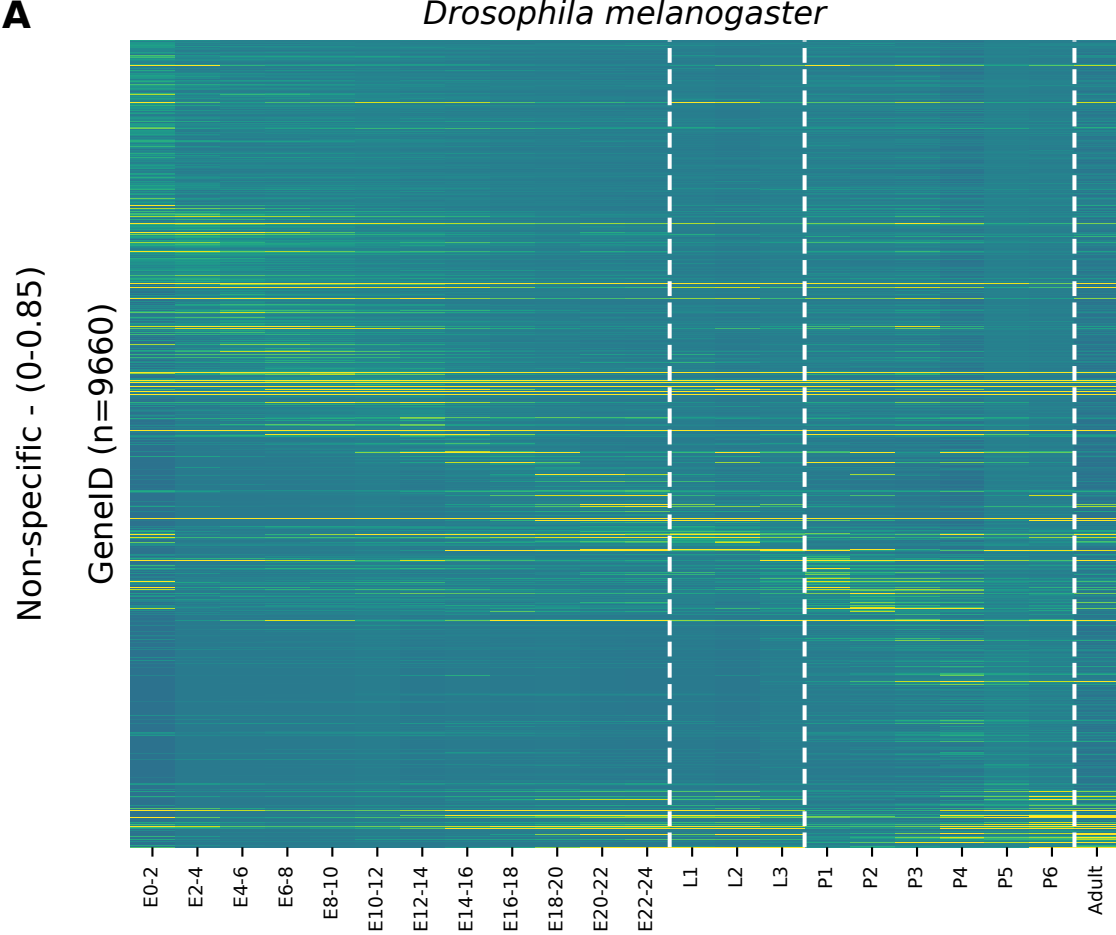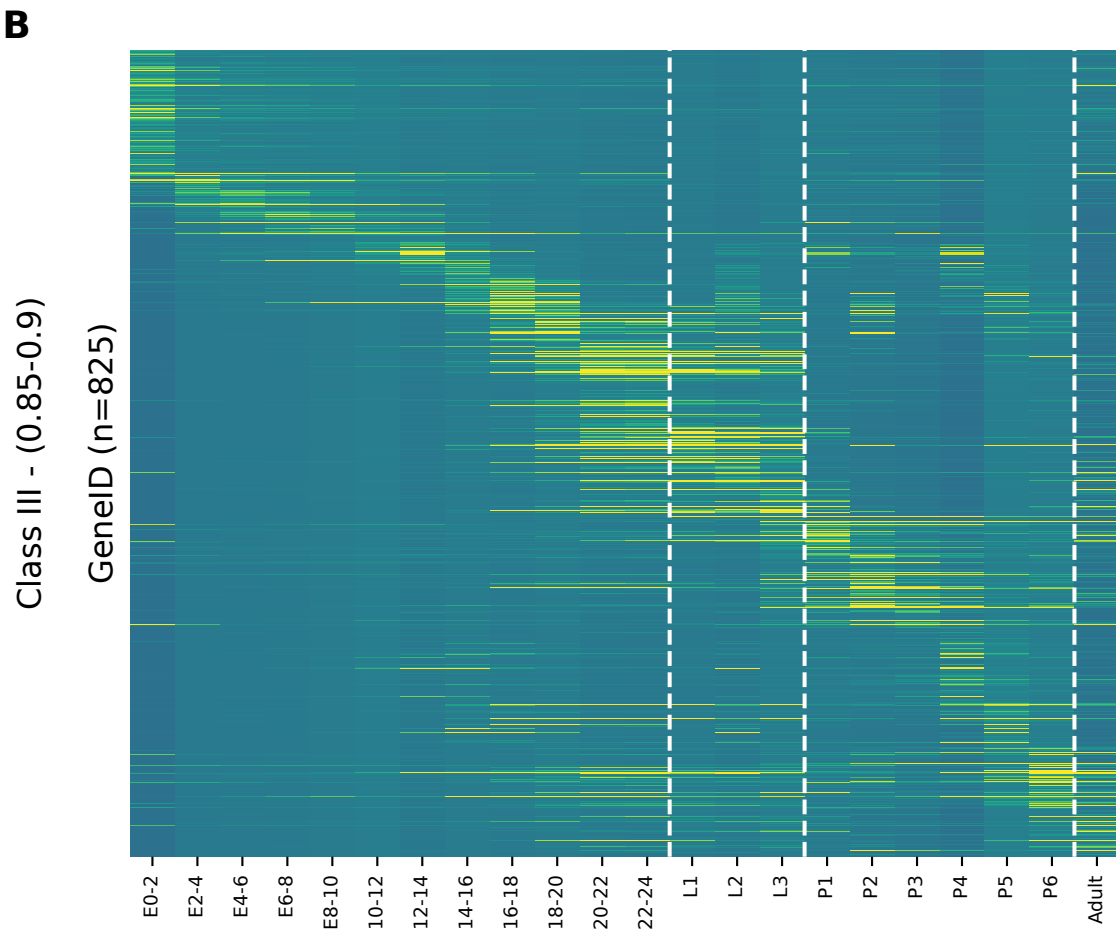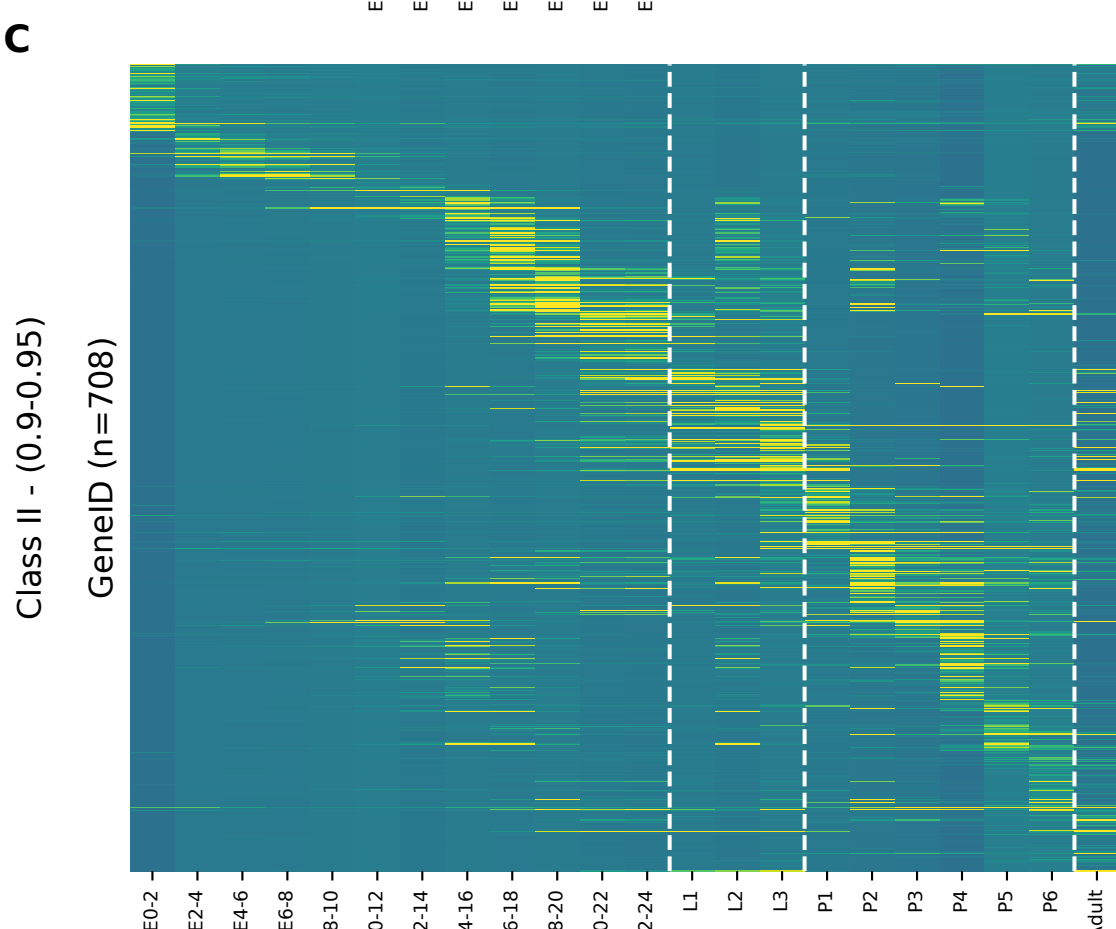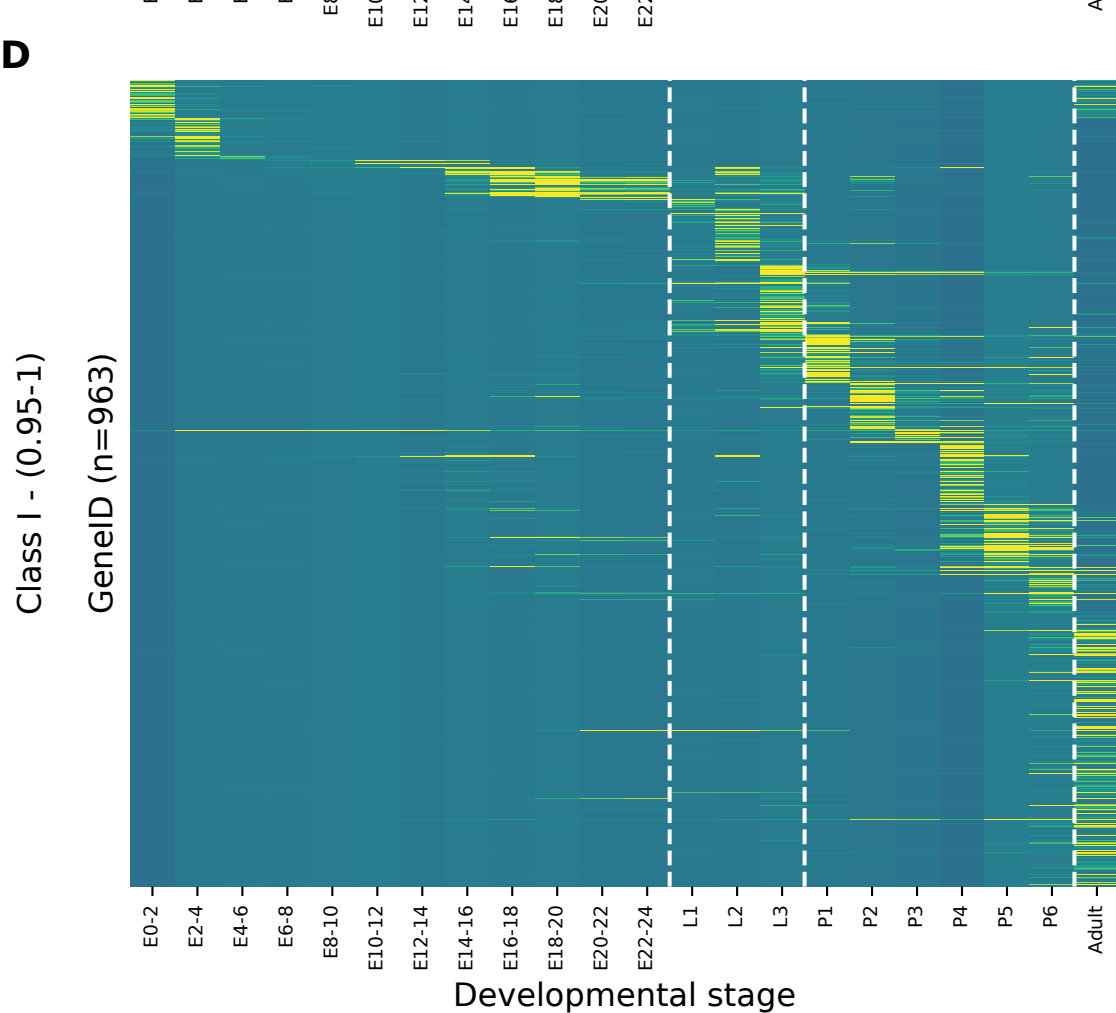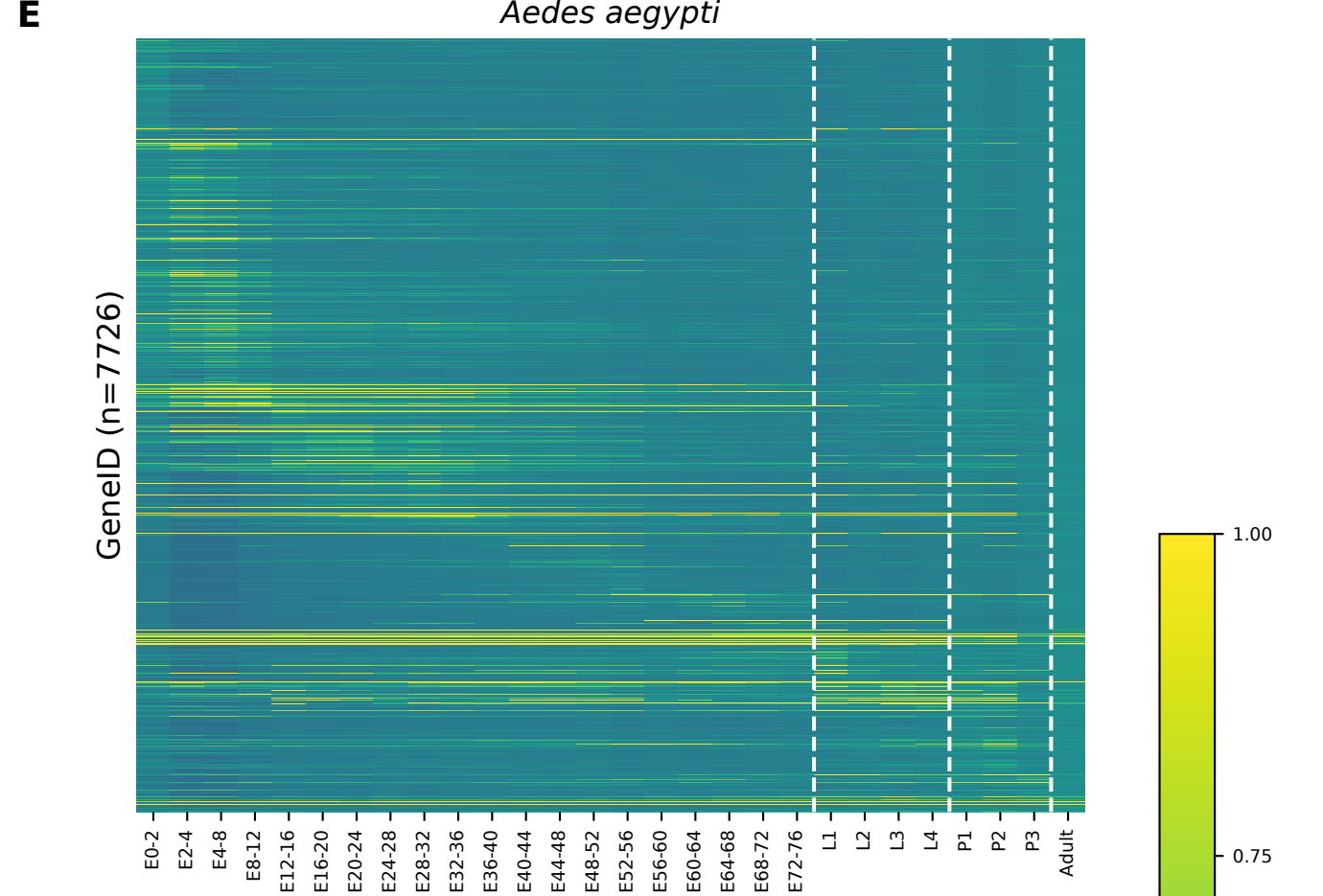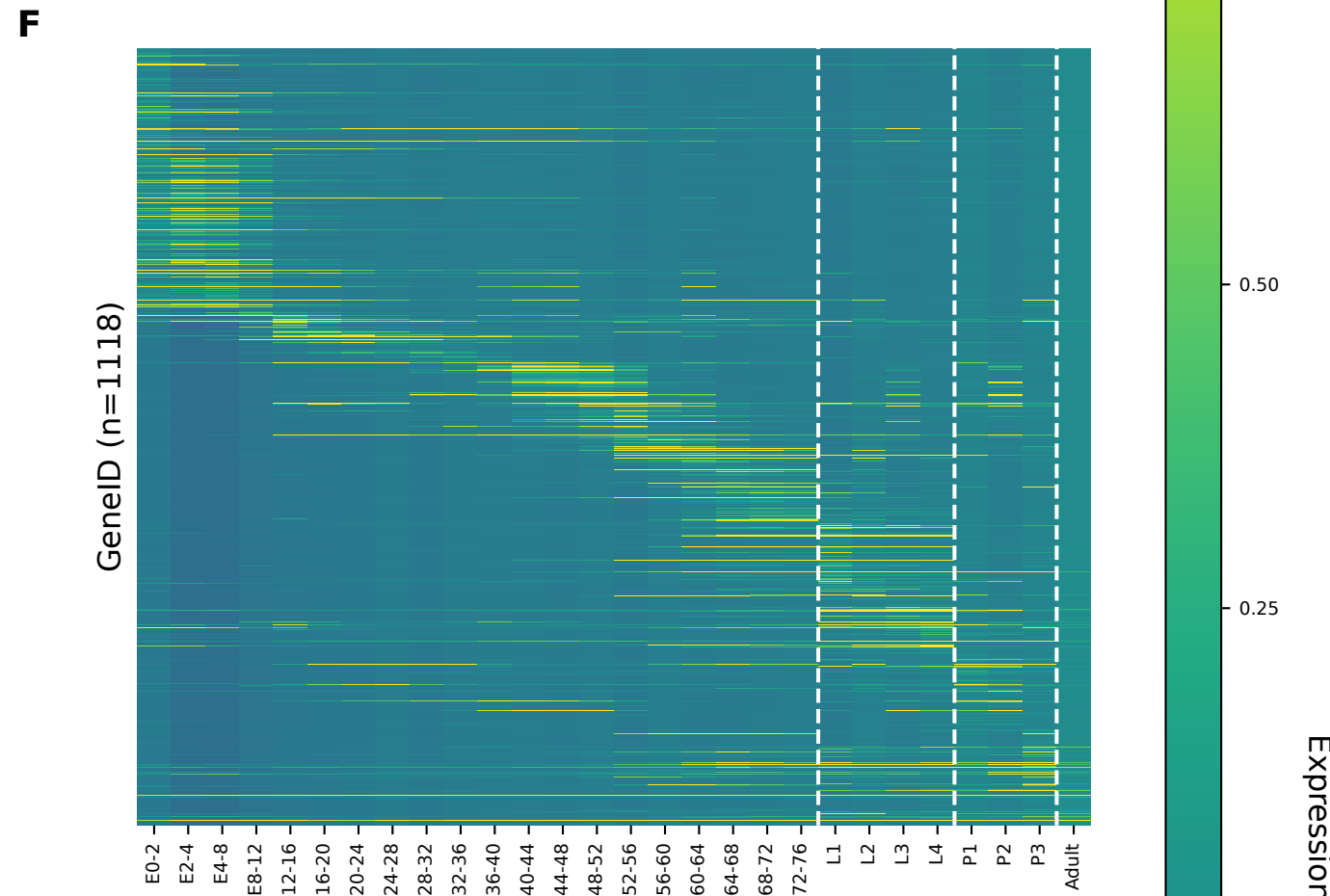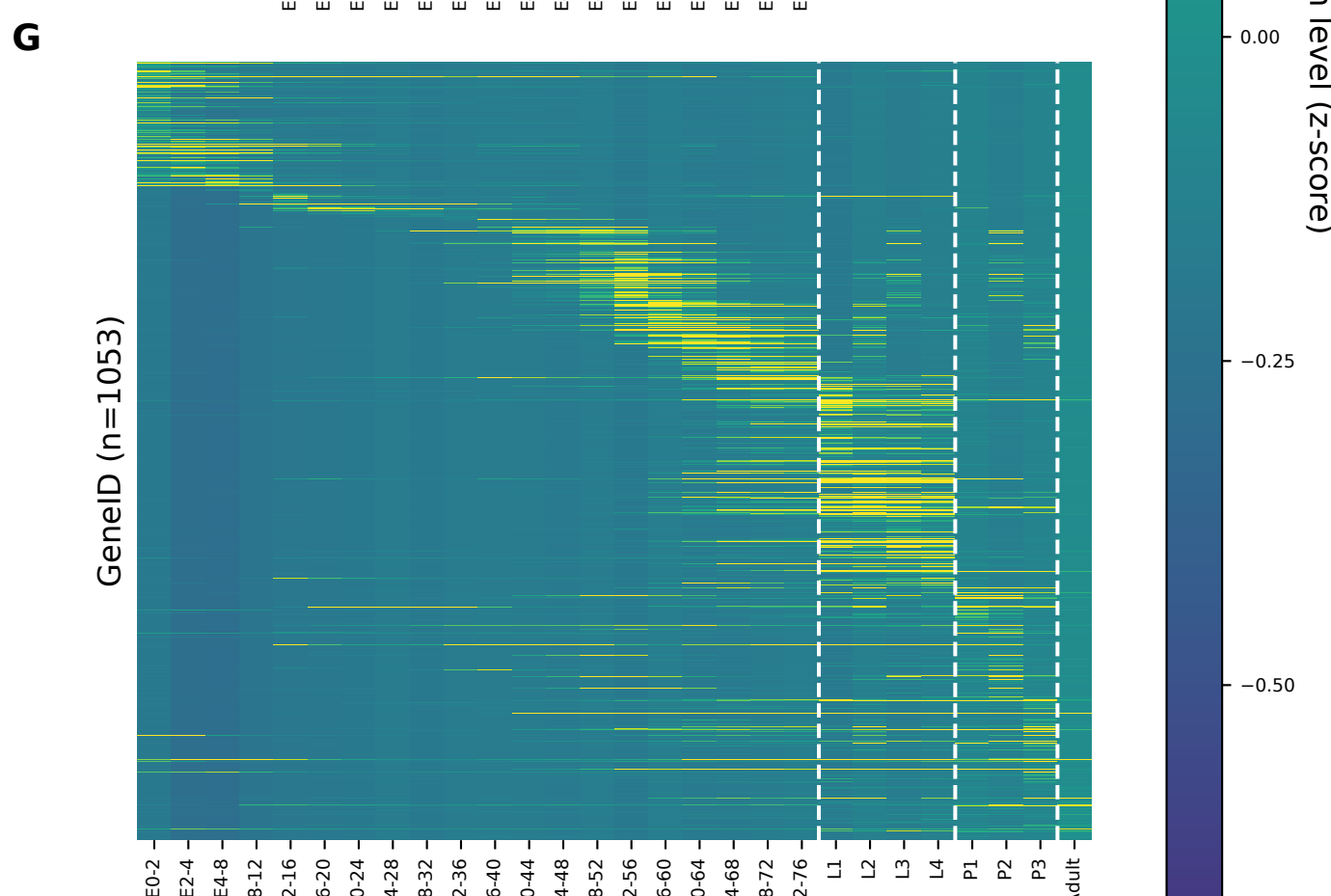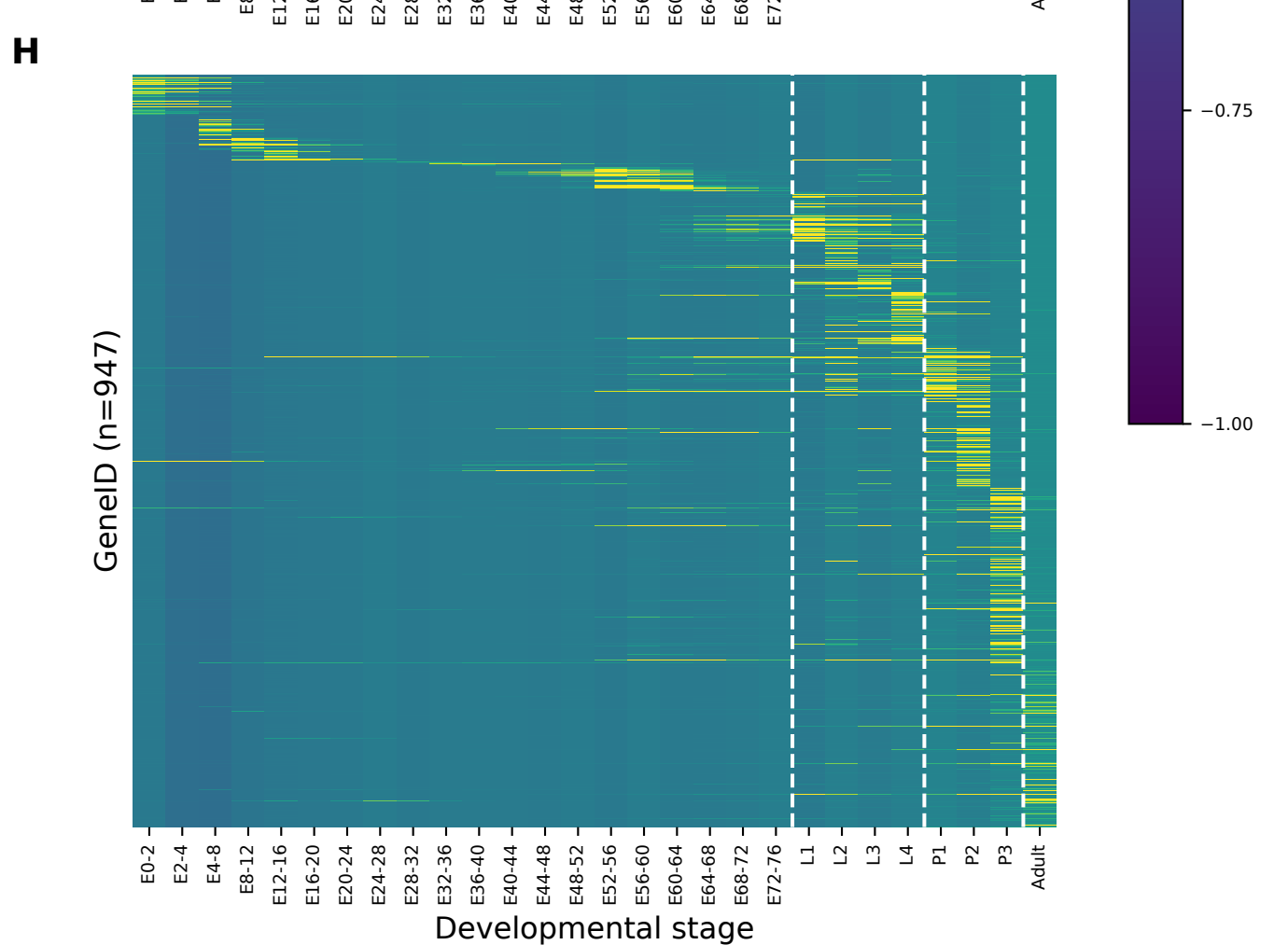

Supplement: Supplementary file 2 — Figure 2 Supplement 1.pdf. [file JEZ-344-428-s007.pdf]

# *Drosophila melanogaster*

**A**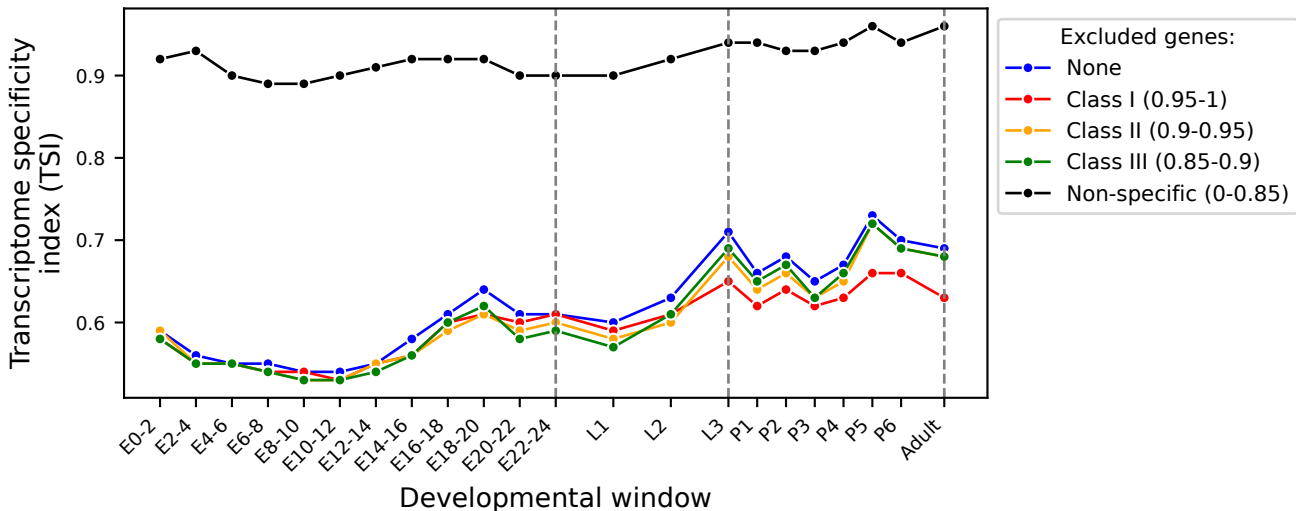

# *Aedes aegypti*

**B**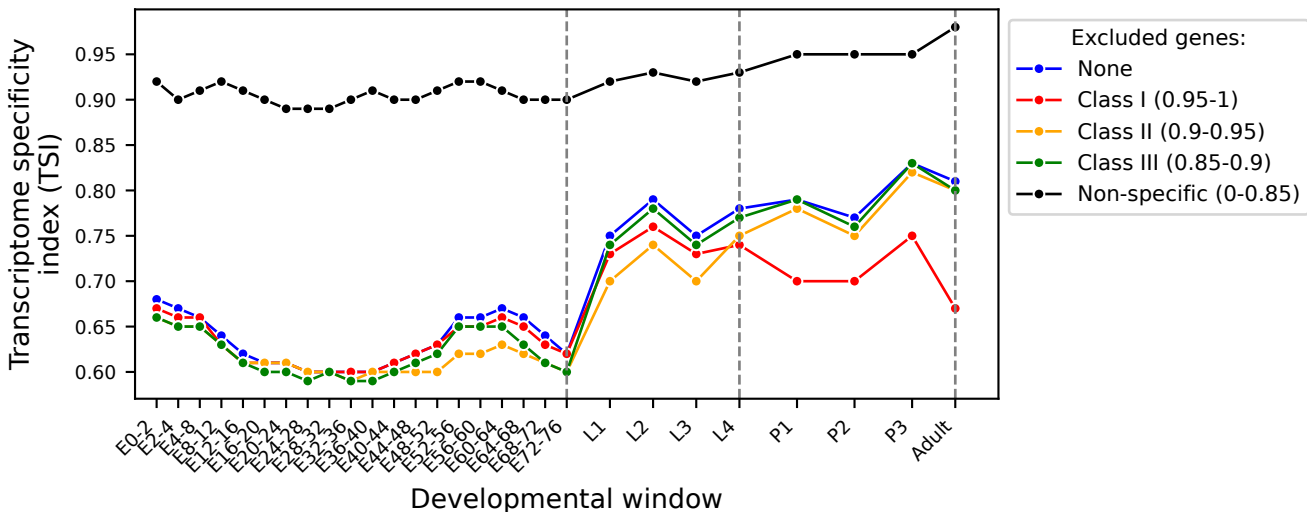

Supplement: Supplementary file 3 — Figure 2 Supplement 1.pdf. [file JEZ-344-428-s001.pdf]

**A**

*Aedes aegypti* genome (1.2Mb windows)

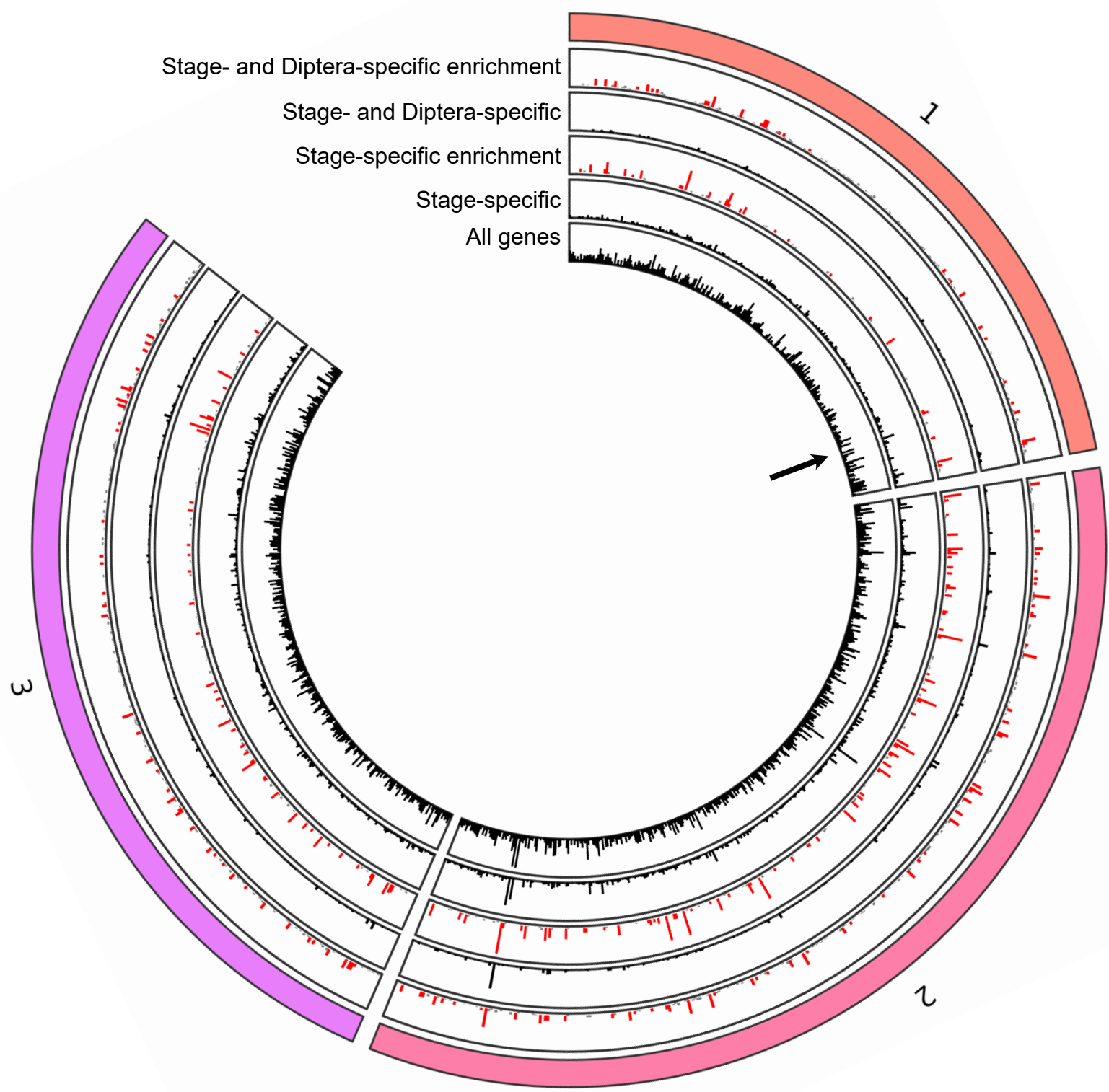

**B**

Enriched window (1.2Mb) on chromosome 2:270000000-271199999

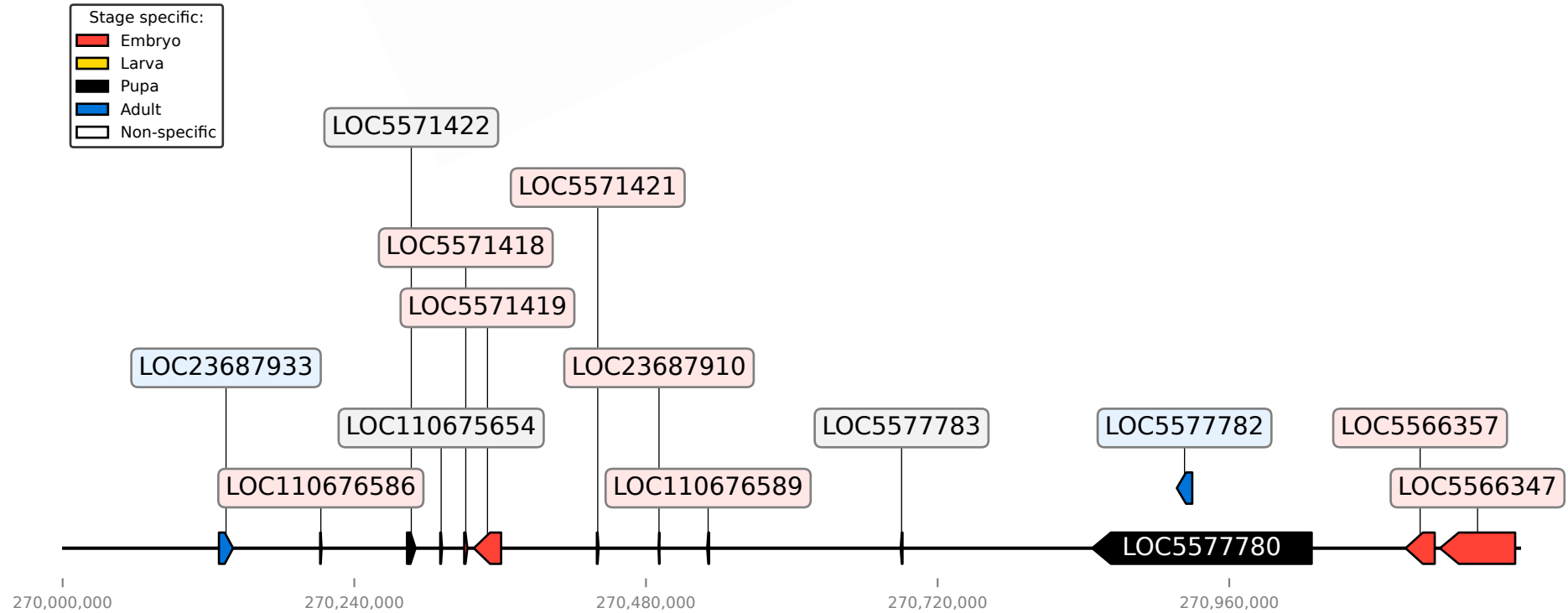

Supplement: Supplementary file 4 — Figure 5 Supplement 1.pdf. [file JEZ-344-428-s009.pdf]
