# Supplementary material for: Recently Evolved, Stage‐Specific Genes Are Enriched at Life‐Stage Transitions in Flies
Source: J Exp Zool B Mol Dev Evol. 2025 Jul 15;344(7):428–41. doi: 10.1002/jez.b.23317 (PMC12576388; doi:10.1002/jez.b.23317)
Supplement: Supplementary file 5 — supmat.docx. [file JEZ-344-428-s010.docx]

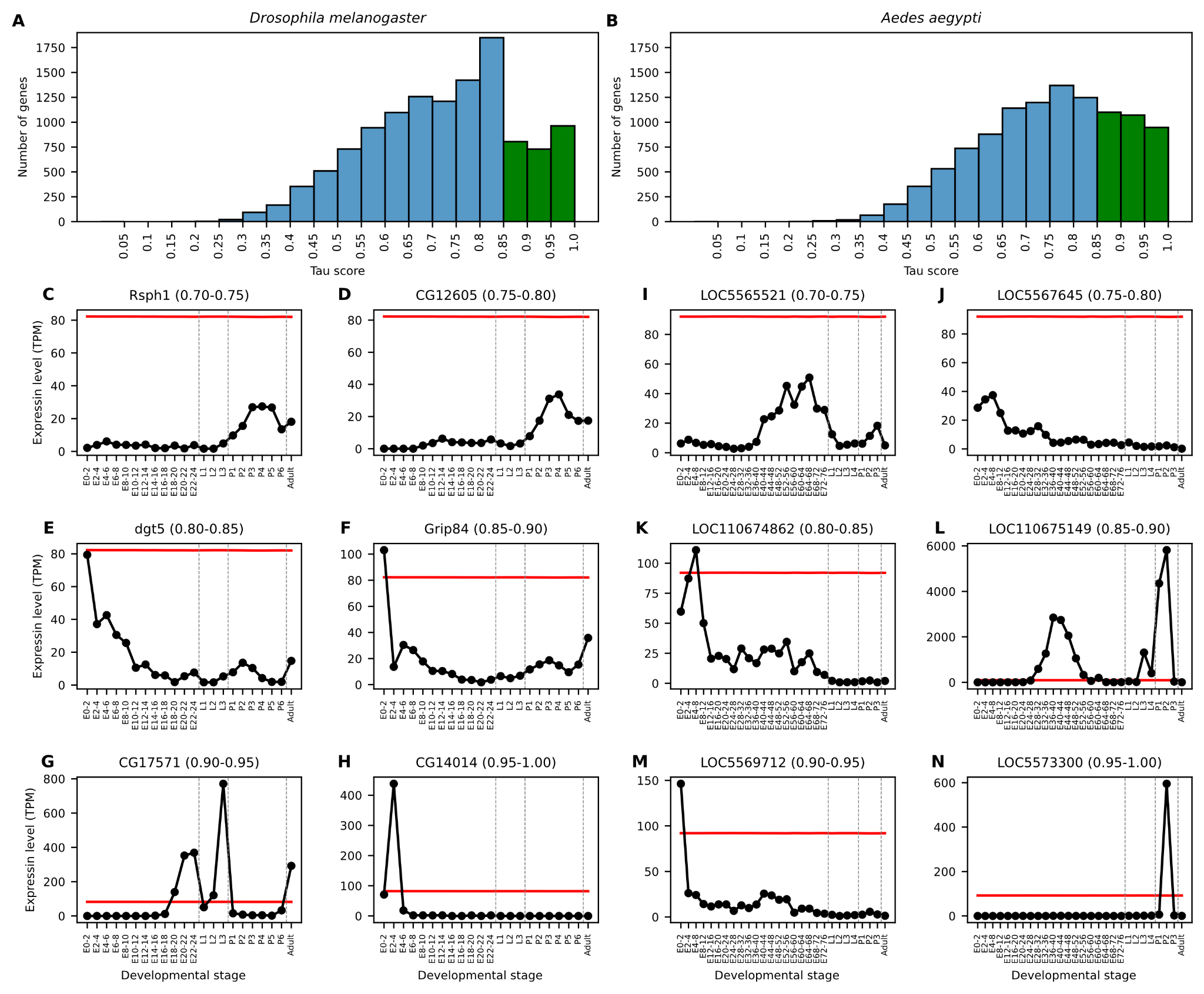


Figure 1 **Supplement 1**: **Classification of stage-specific genes according to tau-score values.** (**A-B**) To score for how strongly transient expression levels were increased during specific stages of development, a tau-score was computed for each gene over the course of development in *D. melanogaster* (**A**) and *A. aegypti* (**B**). Tau scores were based on developmental transcriptomic data that comprised 20 stages in *D. melanogaster*, and 25 in *A. aegypti*. Based on their tau-scores, genes were grouped into 20 bins of 0.05 intervals from 0.00-0.05 to 0.95-1.00. (**C-N**) To illustrate the expression profiles of individual genes comprised in the top six tau-score bins (0.70-0.75, C,I; 0.75-0.80, D,J; 0.80-0.85, E,K; 0.85-0.90, F,L; 0.90-0.95, G,M; 0.95-1.00, H,N), for each bin the temporal expression profile of a randomly picked gene is plotted for *D. melanogaster* (**C** to **H**) and *A. aegypti* (**I** to **N**). FlyBase gene symbols (*D. melanogaster*) and NCBI IDs (*A. aegypti*) and the respective tau-score intervals are indicated on top of each panel. Gene expression levels are normalized as transcript per million (TPM); the red lines mark the mean TPMs computed for all genes expressed in each developmental stage.


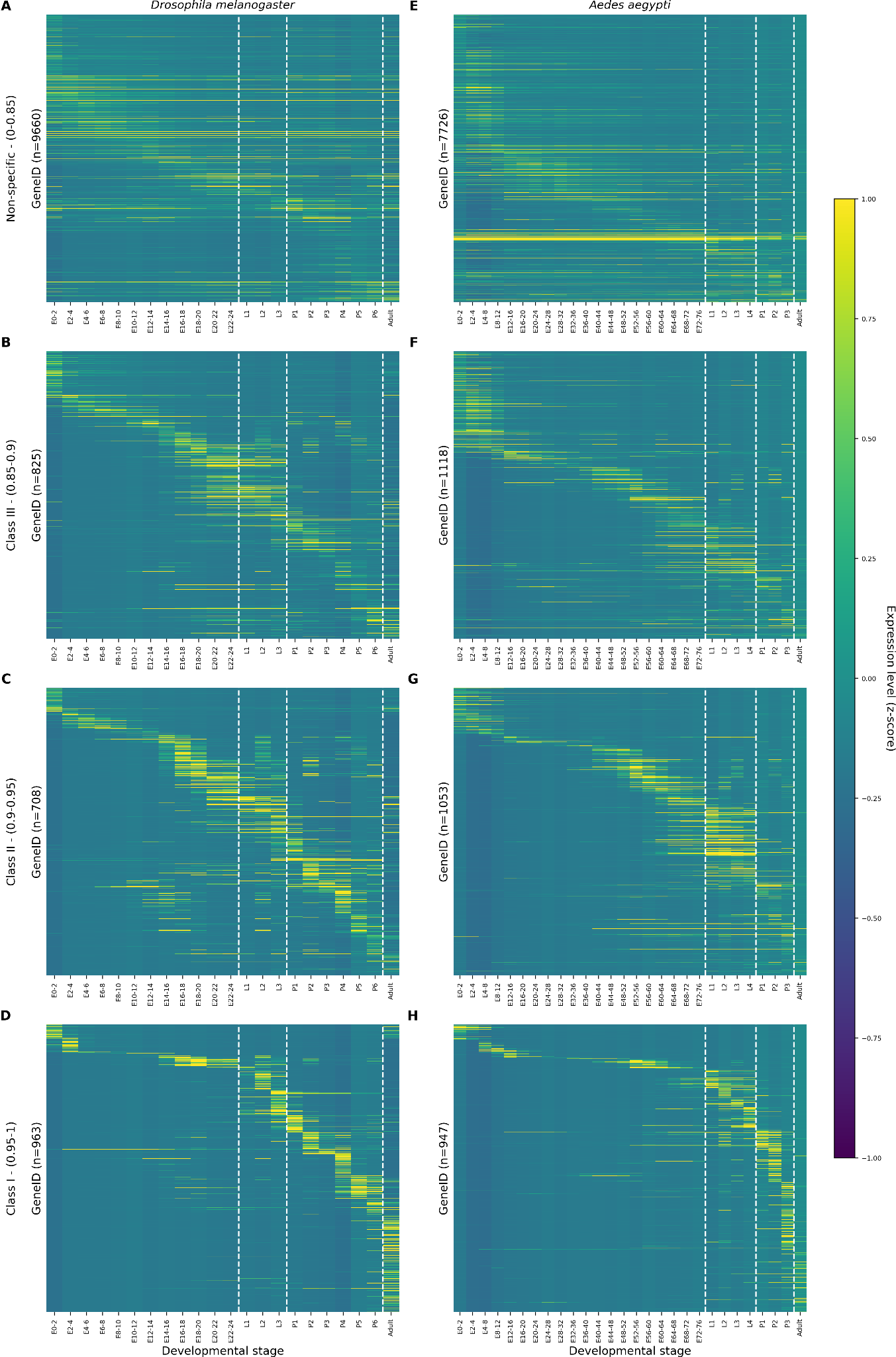


Figure 2 **Supplement 1: Tau-scoring effectively distinguishes genes with single or multiple peaks of expression across developmental stages.** (A-H) All protein-coding genes from *D. melanogaster* (A-D) and *A. aegypti* (D-F) were grouped into four classes of increasing stage specificity (non-specific, 0-0.85; class I, 0.85-0.9; class II, 0.9-0.95; class III, 0.95-1). Most class III genes are expressed transiently in a single developmental stage (D and H), class II genes are mostly expressed in one or two developmental stages (C, G), and class I genes are expressed in up to five developmental stages (B, F). Expression levels for each gene are indicated as standard deviation from its mean expression over the time course of development (z-scores) using heatmap color coding.


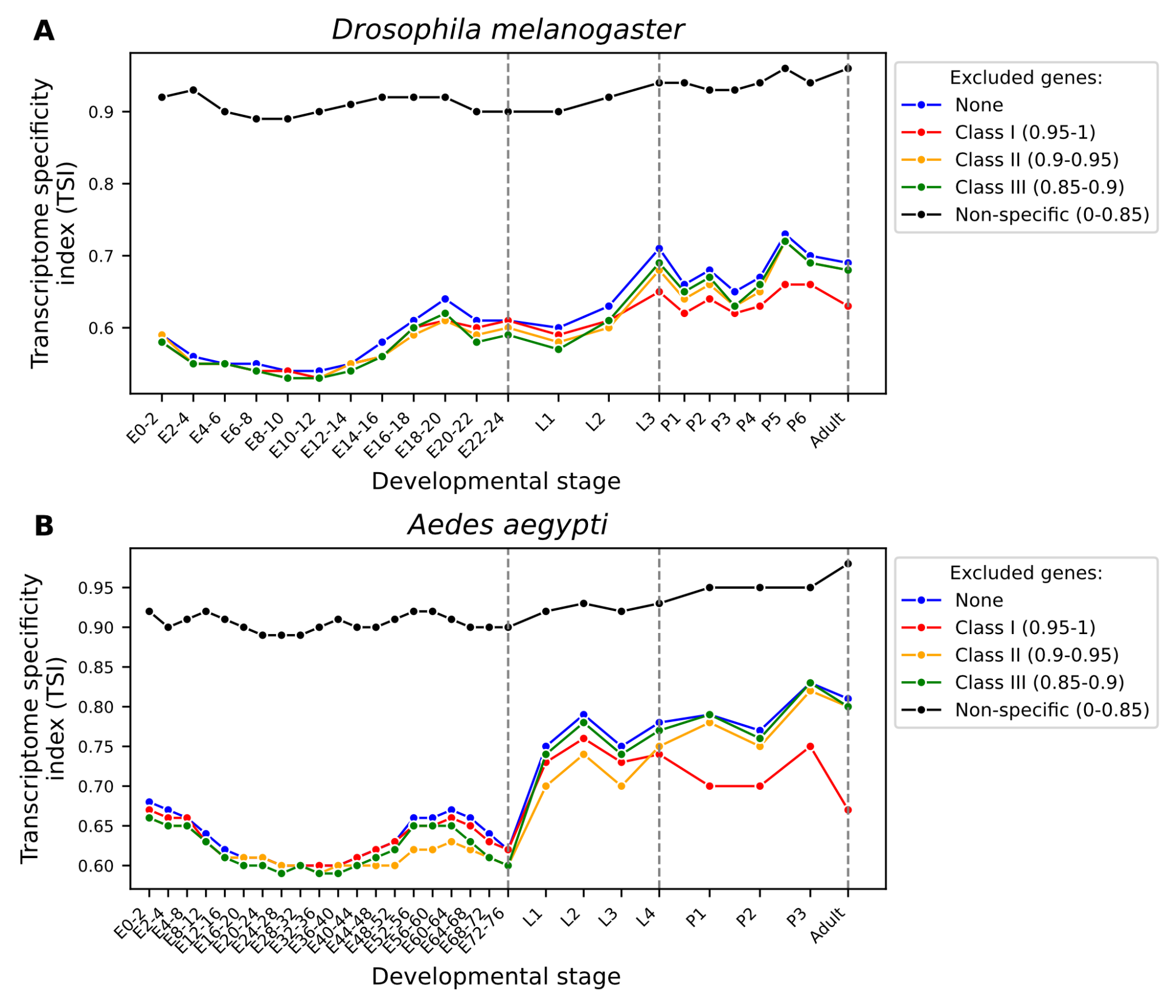


Figure 2 **supplement 2: TSI profiles change when classes of genes with different expression specificity are ​​selectively excluded from the analysis**. (**A,B**) TSI scores were plotted over the course of development in *D. melanogaster* (A) and *A. aegypti* (B), either without any gene class removed (“None”), after removal of class III genes (0.95-1; red), class II genes (0.90-0.95; yellow), class I genes (0.85-0.90; green) or after removal of all non-specifically expressed genes (“Non-specific”, black).


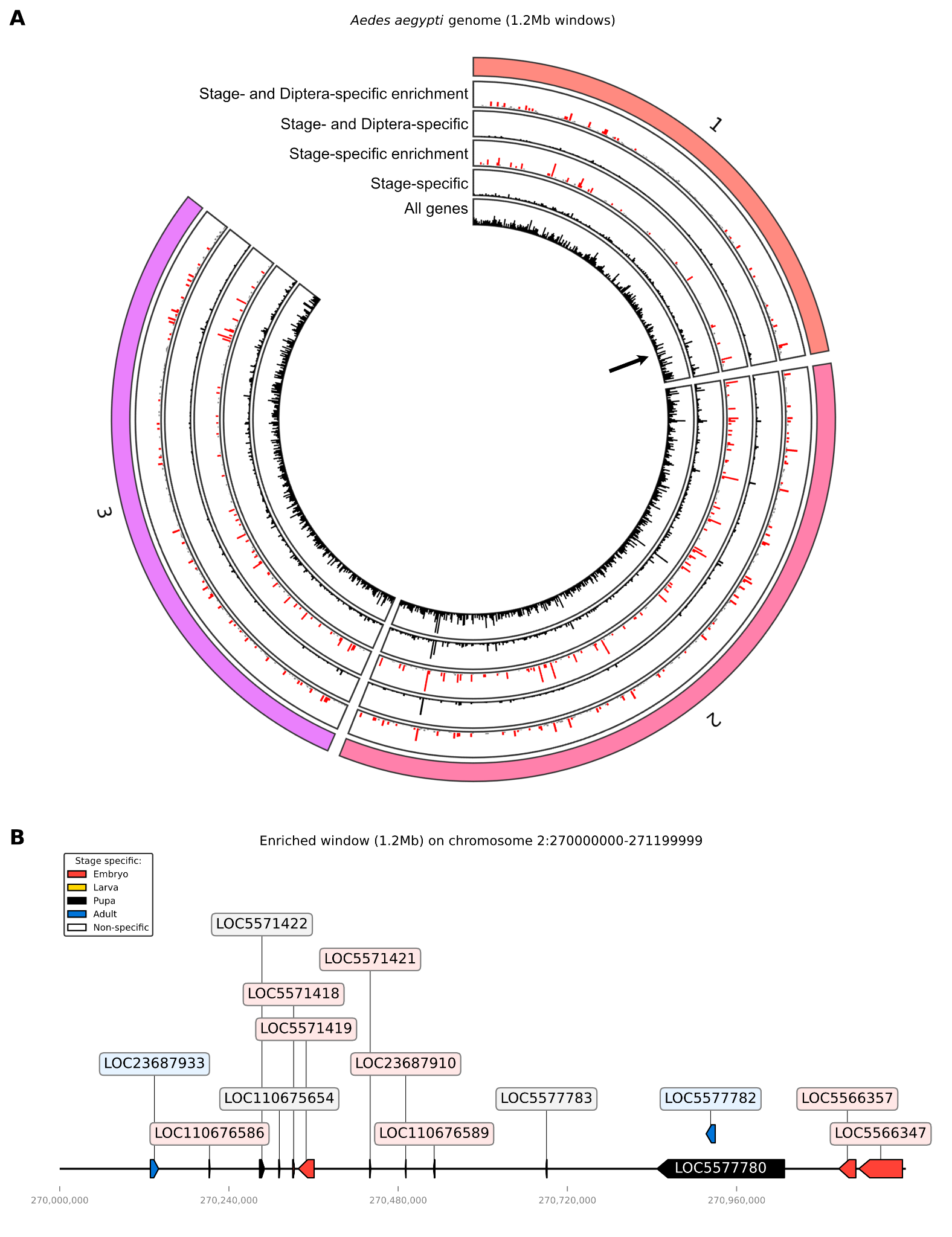


Figure 5 **Supplement 1: Clusters of stage-specific genes in the genome of *A. aegypti*.** (**A**) Based on the average size of TAD, the chromosomes of *A. aegypti* were divided into non-overlapping windows with size of 1200▒kb. Shown for each genomic window are the total number of all genes (innermost ring, black), stage-specific genes (second inner ring, black), the relative enrichment of stage-specific genes (third ring, enrichment in red), the total number of stage-specific genes that originated within Diptera (fourth ring, black), and the relative enrichment of these genes within a given window (fifth ring, enrichment in red). (**B**) Example of a genomic locus with up to 6-fold enrichment of stage-specific genes from chromosome 2 illustrates the clustering of genes in a short genomic interval (the genomic position of the cluster is indicated as a red arrowhead in A; and in Figure 5C as a filled red circle to indicate the outlier).


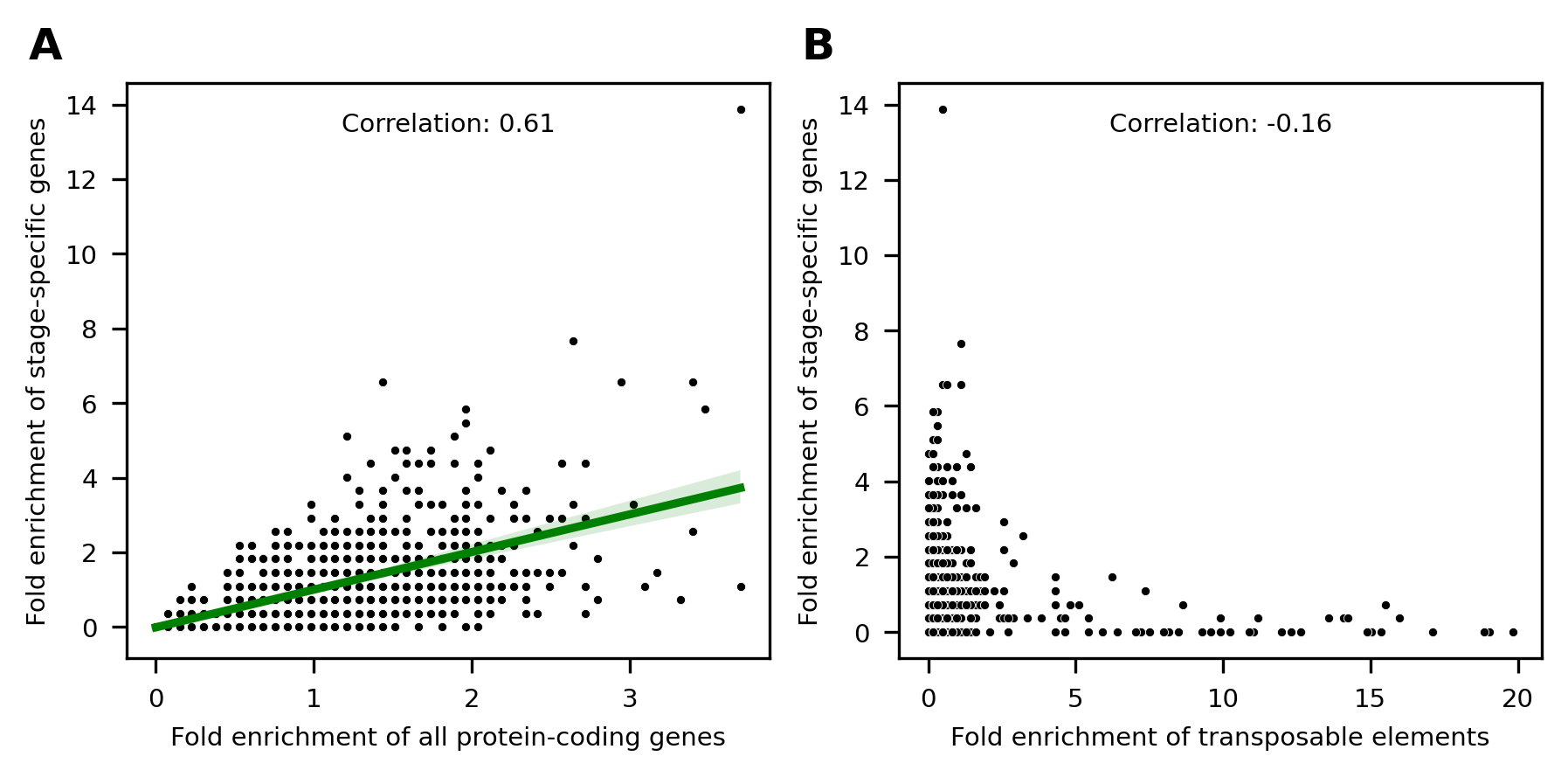


Figure 5 **Supplement 2: Enrichment of stage-specific genes in *D. melanogaster* genomic windows shows moderate correlation with protein-coding genes, but not with transposable elements.** (**A**) The correlation between relative enrichment of protein-coding genes (x-axis) and stage-specific genes (y-axis) across *D. melanogaster* genomic windows (In total of 917 genomic windows). The enrichment of stage-specific genes is in moderate correlation with the enrichment of protein-coding genes (Correlation coefficient 0.61). (**B**) The correlation between relative enrichment of transposable elements (x-axis) and stage-specific genes (y-axis) across *D. melanogaster* genomic windows. The enrichment of stage-specific genes is not correlating with transposable elements (Correlation coefficient -0.16).
